# The Influence of Single Lap Geometry in Adhesive and Hybrid Joints on Their Load Carrying Capacity

**DOI:** 10.3390/ma12121884

**Published:** 2019-06-12

**Authors:** Przemysław Golewski, Tomasz Sadowski

**Affiliations:** Faculty of Civil Engineering and Architecture, Lublin University of Technology, Nadbystrzycka 38, 20-618 Lublin, Poland; pgolewski@gmail.com

**Keywords:** adhesive joints, hybrid joints, cohesive layer, FEM

## Abstract

The manufacturing technology for adhesive joints is not yet fully optimized, as proved by a large number of papers that have been published in recent years. Future studies on innovative techniques for fabricating adhesive joints should investigate the influence of parameters such as: (1) The shape of adhesive protrusion, (2) lap dimensions, and (3) cohesive layer reduction in the most efforted regions of the joint. With the application of additional mechanical connectors (e.g., rivets, screws, and welds) in adhesive joints, new hybrid connections can be fabricated. The number of publications in this new field is still relatively small. To fill the gap, this paper presents the results of a numerical analysis of different single lap geometries in (1) pure adhesive and (2) hybrid joints. A total of 13 different models with the same surface area of the adhesive layer were considered. In the case of hybrid joints, the adhesive surface before the application of mechanical connectors was assumed to be the same in every tested case. The numerical analysis of pure adhesive and hybrid joints revealed that the differences in strength led to a 30% decrease in the load capacity of these joints. Therefore, when designing pure adhesive and hybrid joints, special attention should be paid to the shape of the lap between the joined elements.

## 1. Introduction

Mechanical connections can be formed by welds [1], rivets [2,3], clinch joints [4,5,6], or bolts [7]. Their main disadvantage is a highly concentrated load transfer at fastener points. This causes a very high stress concentration and, moreover, requires the application of hole drilling. These drawbacks can be overcome by introducing an adhesive layer to the connection and thus creating a hybrid joint (HJ) (e.g., [8,9,10,11,12,13,14,15,16,17,18]). The introduction of the adhesive when fabricating clinch joints may have another advantage due to the reduction of friction coefficients between the sheets [13,14]. A proper introduction of the adhesive to the mechanical joint is a difficult process. An example of this can be a spot-welded joint of aircraft skin and stringer in the form of an angle bar [11,18]. In this case, the adhesive must have adequate viscosity and curing time to fill the very narrow space between the stringer and the skin.

On the other hand, pure single-lap adhesive joints (SLJs) under uniaxial tension exhibit significant peel stress concentrations at the ends of the bonded region, which can lead to failure initiation in the connection [19]. These peel stress concentrations can be reduced in a number of ways. They include: Creating an adhesive fillet [20,21,22], changing the adhesive layer thickness or width [23], tapering adherends in the overlap region [20,24], changing the lap geometry by increasing its length [23], using the mixed-adhesive technique [25] to create an adhesive layer with the application of two adhesives with different properties to manufacture functional gradation [26], or fabricating multistep-lap joints [27]. 

Apart from comparing pure adhesive and hybrid joints, future studies should focus on the optimization of the manufacturing technology and modeling of SLJs and other more complex joints of structural elements, e.g., [28,29]. Solutions must be found to assess the influence of different geometric parameters and technological aspects [30,31,32,33,34,35,36]. In general, it is necessary to develop damage tolerant designs of (1) simple joints (SLJs) or (2) more complex structural joints that will have a higher toughness against crack propagation and will be less prone to scatter in strength [37]. Other vital issues include optimization, fracture characterization, and comparisons of FEM models with laboratory results [38,39,40,41].

Future solutions will require more applications for the hybrid joining technique, which entails the use of two or more methods in manufacturing, ideally with a synergistic increase in strength and/or toughness [9]. Hybrid joints have properties that are desired in specific applications for joined structural elements, including: High stiffness and static strength, longer fatigue response and higher amplitude of forces, multi-stage damage and cracking process, higher energy absorption to the final failure, elimination of sealing operations, higher corrosion resistance, enhanced reliability and durability, and reduced manufacturing costs. 

The fundamental challenge for industrial applications is to ensure high reliability and durability of adhesive or hybrid joints over the entire service lifetime of the connected structural elements. The satisfaction of these requirements will lead to optimization and further improvement regarding the following: Enhanced strength and toughness of adhesive joints by the addition of nanoparticles, fibers, or woven mats, e.g., [42,43], or the introduction of a “stop hole” to blunt the tip of a crack [37]; the application of more effective mechanical fastening, e.g., “z-pins” [37]; the modification of the lap joint area geometry; the use of modern joining techniques, such as friction stir welding, e.g., [44], laser beam welding, or electron beam welding.

The strength, damage tolerance, and energy absorption capacity of HJs can be increased in many ways. These include: Finding the optimal overlap size [23,30]; proper surface treatment of the adherend, e.g., [30]; the use of heterogeneous adhesives with silica particles [45] or different nano-reinforcements (graphene flakes or rubber particles); the application of non-flat interfaces or adherend curvature, e.g., [46,47], i.e., the generation of an additional compressive residual stresses to considerably increase load capacity (however, it is more difficult to achieve this geometry and widely use it in complex structural elements); the introduction of a compression to hybrid joints by the use of prestressed mechanical fasteners [8] in order to significantly increase their load capacity, as well as by designing new types of fasteners that allow for adjusting the down force; and, finally, a proper fitting tolerance design for the rivets in HJ holes, which is a very important technological problem [16]. 

The literature on the subject offers very few studies on the effect of geometric and technological parameters in HJ design. In light of the above, the present research will discuss the influence of the shape of the adherend lap with a constant area on the behavior of SLJs and HJs created with the application of rivets. 

## 2. Analyzed Models and Their Designs 

Given numerous papers within an international scope, the problem of single-lap adhesive joints has not lost its relevance. However, to this day there is no wider reference to HJs. The analytical and numerical analyses [15] show that the most strain is carried by the lap edges. Traditionally, the lap edges form straight lines perpendicular to the load direction when the lap has a rectangular shape. Therefore, the aim of the numerical analysis is to determine how the lap shape affects the force value causing damage and further failure of the joint. Different types of lap shapes can be used for repair, e.g., in aviation or sports equipment. Sometimes aesthetic considerations must be satisfied, e.g., by introducing a smooth radius, and at other times, technical aspects must be considered, e.g., through bypassing some elements of the structure. These types of lap shape were developed following consultations with Wit-Composites, a company specializing in the production and repair of laminate products. 

**Figure 1:**
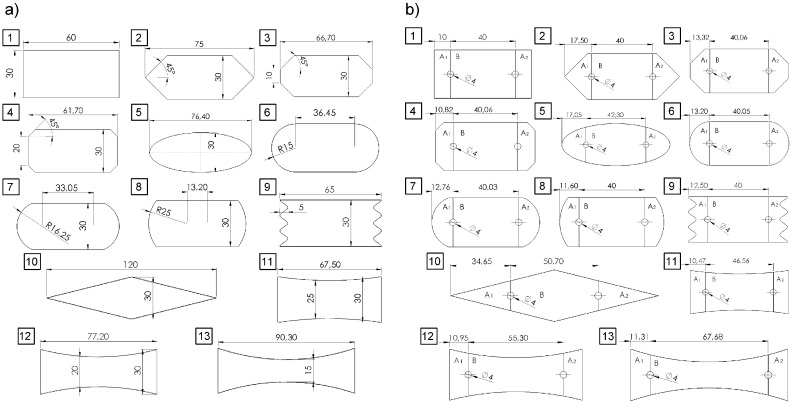
Lap shapes: (**a**) Pure adhesive models, (**b**) hybrid joints. (Length in mm; angle in degree).

**Figure 2:**
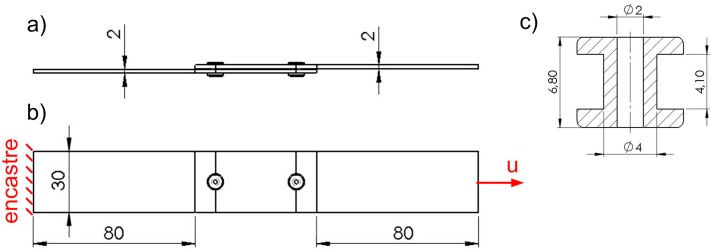
Main dimensions of the reference model (Model 1): (**a**) Side view, (**b**) boundary conditions, (**c**) rivet dimensions. (unit: mm)

For this purpose, 13 different lap shapes were proposed (Figure 1a), for both adhesive and hybrid joints. The most important assumption of this study was that the surface of the adhesive layer would be maintained the same in every tested case. The adherends had a thickness of 2 mm, the joint width was 30 mm, and the adhesive layer thickness was 0.1 mm. Model 1 with a rectangular lap shape (30 mm × 60 mm) was adopted as a reference. The outer diameter of the rivet was 4 mm and the inner diameter was 2 mm (Figure 2). In all cases, the length between the lap edge and the sample end was 80 mm. 

The samples were subjected to uniaxial tensile testing. This meant that every sample was fixed at one end and had a displacement “u” applied to the other end. 

Every adherend was modelled using 23,600 C3D8R elements with four elements through the thickness. C3D8R elements were also used for the rivet model, the mesh of which was made more dense (15,000 elements). The adhesive layer was modelled using 2,113 cohesive elements, COH3D8. A damaged elastic–plastic material model (Mode I fracture) was adopted for 2 mm thick aluminum sheets. The adhesive layer with a thickness of 0.1 mm was modeled with cohesive elements and described with the Mode II fracture of the joint. The numerical analysis was performed using the Abaqus software. The considered material properties are given in Table 1, Table 2 and Table 3.

Due to the presence of mechanical fasteners in the HJ, this model could not be realized as 2D. The only simplification that could be applied was to consider half of the joint along the axis of symmetry. This step, however, was not taken because such models could be used in the future to investigate complex load cases. For a better comparison between pure adhesive and hybrid joints, the first models were realized as three-dimensional, even though many authors [19,20,21] treat them as 2D. However, in the case at hand, the stress distributions in the adhesive layer measured across the width did not suggest that this should be done here (see, e.g., Model 1 just before damage). 

**Figure 3:**
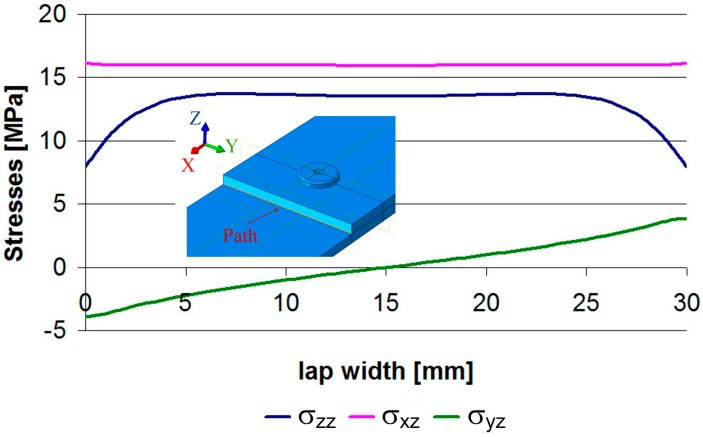
Stresses along the lap width.

Figure 3 shows that only the σ_xz_ stresses have a constant value, whereas the normal stresses σ_zz_ take minimum values at the start and the end of the path, assuming a constant value over a distance of approximately 20 mm. This is due to the fact that the plane strain occurred in the center of the sample, while the plane stress occurred on the side surface. In addition, the shear stresses σ_yz_ changed their sign along the path length, and there was a zero value in the axis. The discussion of stress distribution shows that these connections should not be treated as 2D models. 

## 3. Results Analysis

The proposed models can be grouped as follows:

- Model 1: Reference model to which other models are compared;
- Models 2, 3, 4: Models with 45° chamfers;
- Models 6, 7, 8: Models with different values of the fillet radius;
- Models 11, 12, 13: Models with a lap formed by the arcs.

Other models which cannot be assigned to any of the above groups include: Model 5 with an elliptical lap, Model 9 with pleated lap boundaries, and Model 10 with a diamond-shaped lap. 

**Figure 4:**
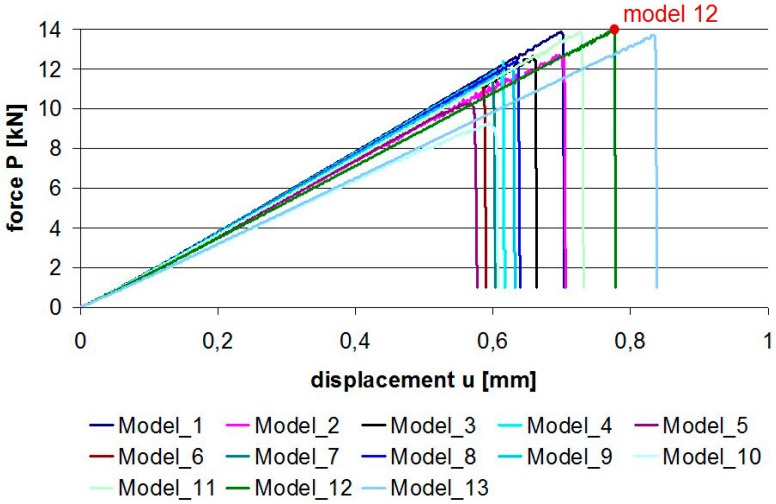
Force–displacement graphs for pure adhesive joints.

Given the maximum force values that can be carried by pure adhesive joints (Figure 4, Table 4), one can draw the conclusion that almost any alteration to the lap shape resulted in a decreased strength of the joint. The only exception was Model 12 which exhibited a slight strength increase amounting to 0.57%. The worst results were obtained for Model 10 in which the strength decreased as much as 33.6%. Therefore, when designing a single lap joint, special care must be taken to ensure that the lap edges form straight and perpendicular lines to the axis of the load. 

**Figure 5:**
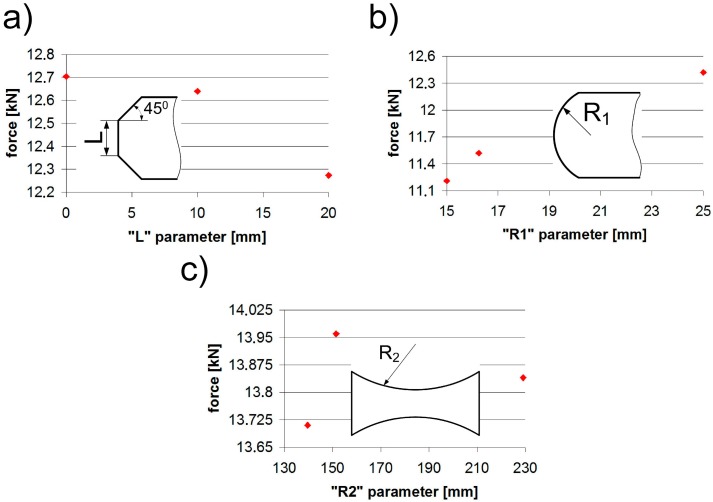
Influence of: (**a**) the chamfer (L), (**b**) the fillet radius (R1), and (**c**) the thinning radius (R2) parameters in pure adhesive joints.

The effects of changing the values of individual shape parameters, i.e., the chamfer (L), the fillet radius (R1), and the thinning radius (R2), are shown in Figure 5 and Table 5. The “L” parameter chart showed that a smaller “L” (bigger chamfer) yielded worse results; however, these differences did not exceed 3.4%. By increasing the “R1” parameter (lap fillet radius), we obtained an increase in strength. In this case, the difference amounted to 9.7%. When analyzing Models 11–13 with the arc parameter “R2” it should be noted that the difference between the lap lengths amounted even to 33.7% (relative to Model 10). Nevertheless, taking into account the changes in force values, the difference was only 1.89%, which made it the least important parameter with respect to the maximum strength.

Regarding HJs, it should be mentioned that the area of the adhesive layer between the mechanical joint axis and the lap edge was maintained the same in every tested case, i.e., A_1_ = A_2_. In all cases the sum of the area was maintained constant (A_1_ + A_2_ + B = const). As a result, in every tested case we observed a difference in length between the rivet axis and the lap edge (Figure 1b). 

**Figure 6:**
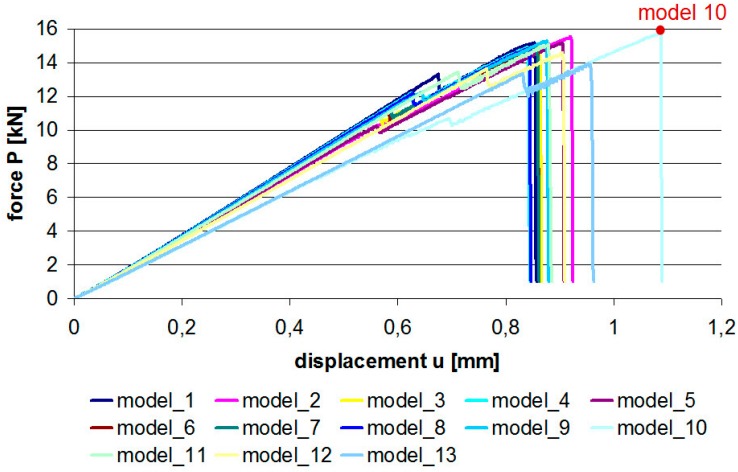
Force–displacement graphs for hybrid joints.

Analyzing the forces obtained for individual joints (Figure 6), one can draw the conclusion that the hybrid joints were much less sensitive to changes in the lap edge shape than pure adhesive joints. In this case, the maximum difference was about 8.2%.

**Figure 7:**
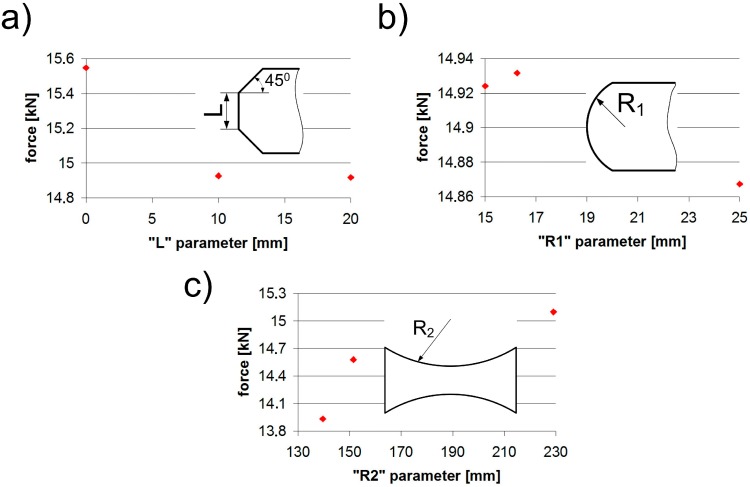
Influence of: (**a**) the chamfer (L), (**b**) the fillet radius (R1), and (**c**) the thinning radius (R2) parameters in HJs.

The influence of individual parameters such as “L”, “R1”, and “R2” is shown in Figure 7. Considering the “L” parameter, it can be observed that a large chamfer was the most advantageous, as the difference was about 3.6%. The second parameter, “R1”, yielded a difference of 0.5%, while the third parameter, “R2”, had the most significant effect with the difference amounting to 8.3%.

**Figure 8:**
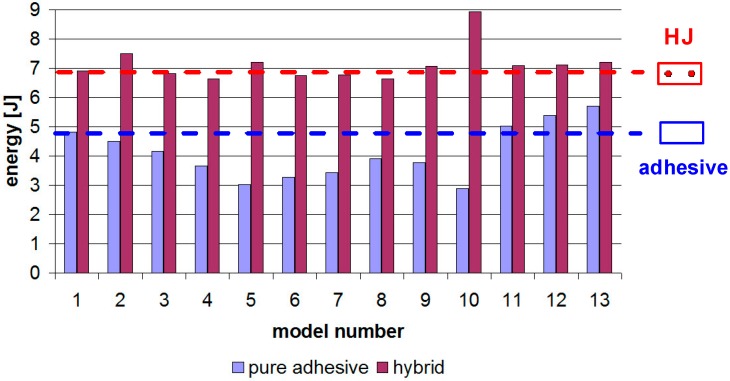
Energy in pure adhesive and hybrid joints.

Figure 8 shows the energy stored in the samples until failure. The HJs were modeled with three-stage damage and cracking processes in:

1. The adhesive layer: Modeled by cohesive elements [3,4,5,6,7,8,9,10,11,12,13,14,15,16,17,18,19];
2. The rivets: The ductile damage model with damage parameters [3,5,8,12,16,48,49,50,51];
3. The adherends: The ductile damage model with damage parameters [3,5,8,12,16,48,49,50,51].

The worst results were obtained for Models 10 and 5, with the differences ranging between 35 and 37%. Importantly, these connections underwent damage with much less displacement at failure, when compared to Reference Model 1. In contrast, the “R2” parameter played an important role. The thinning of the arc-formed central part of the lap led to an increased lap length, joint strength (similarly to [23]), and absorption energy until final failure. This was due to decreased rigidity and the joint may now undergo greater displacements until the adhesive layer is totally damaged. The energy increase in Model 13 was about 18.5%. 

The use of cohesive elements for modeling the adhesive layer allowed us to analyze the time point of damage initiation, in relation to Model 1. This relationship is illustrated in Table 6. One can observe that damage occurred the earliest in Model 10, for which the lowest damage force was also obtained. In all models with the thinning in the central part of the lap, the adhesive layer damage occurred later than in the reference model.

## 4. Laboratory Tests

**Figure 9:**
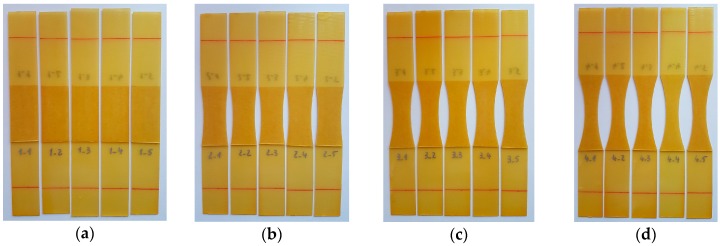
Samples before testing: (**a**) Model 1, (**b**) Model 11, (**c**) Model 12, (**d**) Model 13.

To verify the numerical results, laboratory tests were conducted for one type of connection. Four models were selected for the experiments: 1, 11, 12, and 13. Model 1 was used as a reference. The lap geometries were identical to those in Figure 1a, however, two fundamental changes were made in the construction of the joints. First, a double-sided tape, TESA 51571, was used instead of the epoxy adhesive in order to prevent the influence of flash and to maintain a uniform thickness of the adhesive layer. Both parameters affected the strength of the connection. Second, in the laboratory tests, a linear elastic material was used to eliminate the potential influence of plastic deformation of the adherends. Specifically, a polymer matrix composite (PMC) was used. The reinforcement consisted of six layers of plain fiberglass fabric, and an epoxy resin was used as the matrix. The adherend thickness was set equal to 1 mm. The samples were cut using a water-jet plotter. Prior to the application of the adhesive tape, the sample surfaces were cleaned with the 3M degreaser (surface cleaner sachets). Five samples were made for each model (Figure 9).

**Figure 10:**
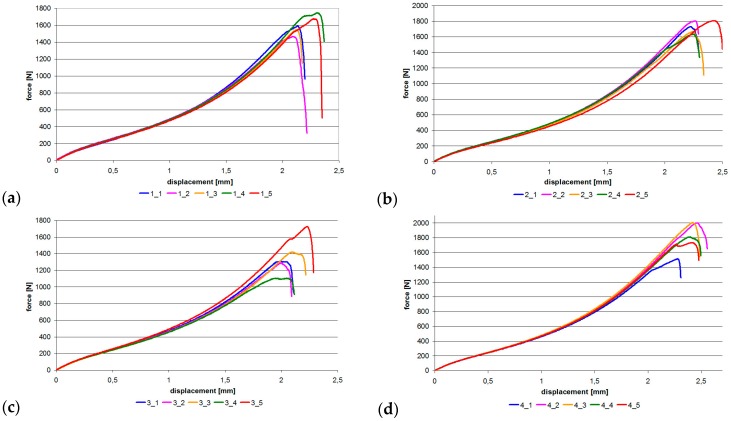
Force–displacement graphs: (**a**) Model 1, (**b**) Model 11, (**c**) Model 12, (**d**) Model 13.

Next, the samples were subjected to uniaxial tensile testing at 2 mm/min using the MTS 25kN testing machine. The first three sample batches underwent damage without audible acoustic effects. Samples from the last batch (Model 13) were damaged with a characteristic crack. The graphs showing uniaxial tensile test results are given in Figure 10. It can be observed that the samples had non-linear characteristics. Once the maximum force was reached, the connection underwent damage. 

The maximum forces and energy leading to damage are listed in Table 7. Naturally, these values were much lower than the results of the numerical analysis wherein the epoxy adhesive was used. Nevertheless, at this point of the study, the percentage relationships between force and energy relative to the reference model were of key importance. 

These relations are presented in Table 8 for changes in the damage energy of individual models with reference to Model 1. For Models 11 and 13, the same trend of energy increase can be observed. This trend was more visible for the experimental models, especially Model 11, wherein the increase was almost double. Regarding Model 13, the difference between the experimental and numerical energy increases was only 4.18%. This experimentally confirmed that the lap shape also affected the strength of the adhesive joint. Only for Model 12 was an opposite trend observed, with reduced strength and damage energy relative to the laboratory reference model.

This trend did not occur in the numerical model, in which the energy increased with the thinning of the central part of the lap. All samples for the laboratory tests were made using the same joining technology. The connections were made carefully using double-sided tape to prevent flash and variations in the thickness of the adhesive layer. One of the reasons for the negative energy obtained in Model 12 could be the hand clamp after joining two halves of the sample. To prevent the influence of pressure, samples for subsequent studies will be made using an adhesive film cured on a press with heated plates.

## 5. Conclusions

In this paper, 26 models of joints (13 pure adhesive and 13 hybrid) were analyzed numerically. The results showed a significant difference between the maximum forces obtained for pure adhesive and hybrid joints. The largest differences exceeding 70% were observed for Model 10, which means that, in a case like this, it is most reasonable to use an additional rivet joint. On the other hand, in the case of Model 13, with thinning in the lap center, the use of additional mechanical connectors is not advisable because the force increase was only 1.6%.

The failure of hybrid joints took place in two stages. In the first stage, the adhesive layer was damaged to the rivet boundary, which was indicated by a slight decrease in force. In the second stage, the force increased until damage of the mechanical and adhesive joints occurred. This phenomenon can be used to monitor the connection status, e.g., by using a strain gauge or piezoelectric sensor that will react to a decrease in strength and trigger an alarm procedure. 

Other conclusions that can be drawn from this research were as follows:

- The chamfer parameter “L” had a similar effect in pure adhesive and hybrid joints alike, its value ranging from 3.4% to 3.6%;
- The fillet parameter “R1” had a significant effect only in pure adhesive joints, amounting to 9.7% for pure adhesive joints and to 0.5% for hybrid joints;
- The opposite was observed for the “R2” parameter, which was insignificant in pure adhesive joints (1.8%), but its effect on energy amounted to 8.3% in hybrid joints;
- In the case of Model 13, the difference between the experimental and numerical energy increased values was only 4.18%.

## Figures and Tables

**Table 1:** Material properties of aluminum plates.

Yield Stress σ_y_	Tensile Strength σ^t^	Elongation at Break *A*	Young’s Modulus *E*	Poisson’s Coefficient *ν*
470 MPa	540 MPa	0.08	70 GPa	0.3

**Table 2:** Material properties of aluminum rivets.

Yield Stress σ_y_	Tensile Strength σ^t^	Elongation at Break *A*	Young’s Modulus *E*	Poisson’s Coefficient *ν*
400 MPa	450 MPa	0.5	70 GPa	0.3

**Table 3:** Material properties of epoxy adhesive [24].

Young’s Modulus *E*	Kirchhoff’s Modulus *G*	Shear Strength *τ*_u_	Normal Strength σ_u_	Fracture Energy *G*_I_
0.1 GPa	0.038 GPa	25 MPa	18 MPa	200 J/m^2^

**Table 4:** Failure force of adhesive joints.

Model	1	2	3	4	5	6	7	8	9	10	11	12	13
Failure force (kN)	13.88	12.70	12.64	12.27	10.26	11.22	11.53	12.42	12.01	9.25	13.85	13.96	13.71

**Table 5:** Failure force of hybrid joints (HJ).

Model	1	2	3	4	5	6	7	8	9	10	11	12	13
Failure force (kN)	15.18	15.55	14.93	14.92	15.15	14.92	14.93	14.87	15.28	15.78	15.10	14.58	13.93

**Table 6:** Time of damage initiation and final failure, expressed in percentages, in relation to Reference Model 1.

Model	1	2	3	4	5	6	7	8	9	10	11	12	13
Start (%)	100.0	72.9	82.9	82.1	74.3	82.9	85.0	90.7	64.3	47.1	103.6	110.7	119.3
End (%)	100.0	100.0	94.3	87.1	81.4	84.3	85.7	90.7	90.0	86.4	104.3	110.7	119.3

**Table 7:** Maximum force and energy.

	Sample 1	Sample 2	Sample 3	Sample 4	Sample 5	Mean Value
Model 1						
Max force (kN)	1.59	1.47	1.55	1.75	1.68	1.60
Energy (J)	1.45	1.42	1.37	1.74	1.63	1.52
Model 11						
Max force (kN)	1.73	1.80	1.67	1.64	1.81	1.73
Energy (J)	1.60	1.64	1.62	1.60	1.86	1.67
Model 12						
Max force (kN)	1.31	1.29	1.42	1.11	1.73	1.37
Energy (J)	1.23	1.18	1.36	1.15	1.62	1.31
Model 13						
Max force (kN)	1.51	2.00	2.01	1.81	1.73	1.81
Energy (J)	1.51	2.08	1.98	1.91	1.87	1.87

**Table 8:** Results comparison.

	Model 11	Model 12	Model 13
Experiment	9.49%	−13.96%	22.64%
FEM	4.35%	11.61%	18.46%
